# The effectiveness of remote delivered intervention for loneliness reduction in older adults: A systematic review and meta-analysis

**DOI:** 10.3389/fpsyg.2022.935544

**Published:** 2022-07-28

**Authors:** Zhengke Fu, Mengsi Yan, Chao Meng

**Affiliations:** ^1^Department of Computational Communication, School of Media and Law, Ningbo Tech University, Ningbo, China; ^2^Department of Japanese Language Study, School of Humanity, Ningbo University of Finance and Economics, Ningbo, China

**Keywords:** remote delivered intervention, loneliness, older adults, meta-analysis, systematic review

## Abstract

**Background:**

Remotely delivered intervention is widely applied to loneliness treatment in older adults, but the effect is controversial. This study aimed to evaluate the effects of remotely delivered intervention on loneliness using a systematic review and meta-analysis.

**Methods:**

The PubMed, the Cochrane Central Register of Controlled Trials, EMBASE, CINAHL (EBSCO), PsycINFO (EBSCO) databases were searched for studies, the search ended on 7 July 2021. Thirteen randomized controlled trials of remotely delivered intervention compared with usual care, brief contact, or no intervention for loneliness were included. A random-effects model measured estimation of loneliness reduction. Furthermore, standardized mean differences (SMDs), 95% confidence intervals (CIs), publication bias, and heterogeneity were calculated. Subgroup analysis was used to explore the factors that might affect the treatment effects.

**Results:**

The evidence of remotely delivered intervention on loneliness reduction was certain (SMD = −0.41 [95% CI, −0.70 to −0.13]). Media subgroup analysis supported the effectiveness of intervention delivered by video call (SMD = −0.54 [95% CI, −0.83 to −0.25]); treatment strategy subgroup analysis found evidence to support the effectiveness of increasing social support and maladaptive social cognition treatment strategy (SMD = −0.47 [95% CI, −0.77 to −0.18] and SMD = −1.04 [95% CI, −1.98 to −0.10], respectively); participants subgroup analysis shown the effectiveness of intervention for older adults living in LTC and social isolation (SMD = −1.40 [95% CI, −2.43 to −0.36] and SMD = −0.55 [95% CI, −0.74 to −0.36], respectively); group format subgroup analysis testified the effectiveness of intervention carried out in individual format (SMD = −0.39 [95% CI, −0.71 to −0.07]); measurement time points subgroup analysis found the positive effect of intervention at 3 months and 3 to 6 months stage (SMD = −0.33 [95% CI, −0.52 to −0.14] and SMD = −0.32 [95% CI, −0.57 to −0.07], respectively). Significant publication bias was detected (*p* < 0.05), and the heterogeneity of the studies was substantial.

**Conclusion:**

This systematic review and meta-analysis demonstrate that remotely delivered intervention can reduce loneliness in older adults, and it appears to be affected by media type, treatment strategy, participants characteristics, group format, and measurement time points.

## Introduction

Loneliness is common among older adults, and it can have side effects such as social isolation (Mountain et al., 2014), depression (Heller et al., 1991), less social support (Choi et al., 2020), and lead to suicide (Conwell et al., 2021). The World Health Organization is estimated to have 20–40% of affected older adults (Perissinotto et al., 2012). The main therapeutic goal in the treatment is loneliness reduction.

Loneliness interventions are based on four strategies: (a) enhancing social skills; (b) providing social support; (c) increasing opportunities for social interaction; and (d) addressing maladaptive social cognition (Masi et al., 2011). These intervention elements could be adapted for remote delivery (Yousefi Nooraie et al., 2021). In mental health problem treatment, the satisfaction of remotely delivered intervention is equivalent to or significantly higher than face-to-face intervention (Guaiana et al., 2021).

Because many older adults with loneliness have trouble accessing support groups or specialists for physical conditions or traffic barriers (Chesney et al., 2003; Crystal et al., 2003), remotely delivered intervention may be a practical option in treating loneliness (Poscia et al., 2018). Information and communication technology may overcome the social and spatial barriers of social interaction by enabling accessible, affordable communication and activities of multiple forms (i.e., textual, audio, or visual) between the elderly (often with limited mobilization) and others anytime and anywhere (Chen and Schulz, 2016). With Corona Virus Disease 2019 (COVID-19) shielding/social distancing measures, remotely delivered interventions for older adults become more urgent (Williams et al., 2021).

Remotely delivered intervention could include psychology and sociology intervention (Gorenko et al., 2021). Although not fully understood, the theoretical psychological basis of intervention is considered to include the change in social behavior by changing persons’ mental process. For example, cognitive behavioral therapy helps individuals to look for disconfirming evidence to reframe perceptions of loneliness and self-efficacy to change behaviors, increase social connections, and decrease loneliness (Hickin et al., 2021). The theoretical sociological basis of intervention includes the increase of social engagement by connecting to the outside world, improving social skills, engaging in activities of interest, and boosting self-confidence. Increased social engagement is linked to decreased risk of cognitive decline, depression, and loneliness (Gorenko et al., 2021). For each patient, information on participants’ technology accessibility and needs are used to define the proper media type and treatment strategy to achieve optimal therapeutic effects (Gorenko et al., 2021).

Effects of entirely remotely delivered interventions have been evaluated on different mental illnesses, like depression (Guaiana et al., 2021), schizophrenia (Kasckow et al., 2014), mental disorders (Leach and Christensen, 2006), psychotic disorders (Baker et al., 2018), and other mental health problems (Hailey et al., 2008). Whether remotely delivered interventions have a definite therapeutic effect on loneliness in older adults is controversial (Ibarra et al., 2020). Because of the small number of published studies and their heterogeneity, many systematic reviews have reported inconsistent results (Masi et al., 2011; Choi et al., 2012; Cohen-Mansfield and Perach, 2015; Gardiner et al., 2018; Noone et al., 2020; Jin et al., 2021; Lee et al., 2021; Williams et al., 2021). Nevertheless, several high-quality randomized controlled trials (RCTs) have recently been published (Lai et al., 2020; Kahlon et al., 2021; Shapira et al., 2021). This study aimed to conduct an updated meta-analysis and systematic review on the loneliness reduction obtained by remotely delivered intervention for loneliness in older adults.

## Materials and methods

### Protocol and registration

We followed the reporting guidelines of the Preferred Reporting Items for Systematic Reviews and Meta-Analysis 2020 (PRISMA 2020; Page et al., 2021). The completed PRISMA 2020 checklist was provided in online Supplementary material. The protocol was registered in PROSPERO (registration number is CRD42021285534).

### Search strategy

We identified studies that evaluated the efficacy of telephone-delivered intervention for older adults with loneliness by searching the following electronic databases: PubMed, the Cochrane Central Register of Controlled Trials, EMBASE, CINAHL (EBSCO), PsycINFO (EBSCO). The search ended on 7 July 2021. A combination of free-text terms and medical subject heading terms was used for the subject search. Search terms included the following: (1) aged, aging, elderly, old*; (2) lone*; combined with (3) telephone and hotline. Using PubMed search strategy as an example, the detailed search strategy in online Supplementary material. After the electronic search, we supplementary screened relevant articles from the reference lists of included studies or previous systematic reviews. The language of included studies was English.

### Inclusion criteria

Studies were included based on the following criteria: (1) older adults who are over the age of 65 years (Shenkin et al., 2017), whether or not they were experiencing symptoms of loneliness, social isolation, depression, anxiety, or other mental illness at baseline; (2) treatment by remotely delivered intervention; (3) treatment of a control group with brief contact, social activity, usual care or no intervention; and (4) outcomes of loneliness as measured with any instrument.

### Definitions

For the meta-analysis, the remotely delivered intervention group comprised the patient who received any intervention delivered *via* the telephone, video call, internet, or computer, with a social connection or psychosocial (mental, emotional, social, or spiritual) focus, or a combination of these. And the control group included those who received brief contact, usual care, or no intervention, “brief contact” represented brief calls (Lai et al., 2020) or brief telephone visits (Choi et al., 2020), “social activity” represented sports activity (Jing et al., 2018) or daily social activity (Shapira et al., 2021), “usual care” represented standard, conservative therapy (Kahlon et al., 2021), “No intervention” represented either no routine treatments or alternatives (Tsai et al., 2020).

### Data extraction

Two authors (FZ and YM) independently reviewed all titles and abstracts to determine eligibility and retrieve articles. Two authors resolved their disagreement by discussion. If they could not make an agreement, another author (MC) was consulted, and a decision was made by a majority vote. The following information was extracted based on a fixed protocol: authors, year of publication, country, age distribution, gender proportion, study design, numbers of remotely delivered intervention and control participants, the intervention and control groups (e.g., intervention media, strategy, group format, participants’ background, duration of follow-up), measurement time points(s) and outcome measures.

### Validity assessment

As described in the Cochrane Handbook for Systematic Reviews of Interventions, the Cochrane Collaboration’s risk of bias tool was used to assess bias in each eligible study (Higgins and Green, 2008). The quality assessment covered the following domains: (1) sequence generation; (2) allocation concealment; (3) blinding; (4) incomplete outcome data; (5) selective outcome reporting; and (6) other possible sources of bias. The meta-analysis results were interpreted in terms of findings regarding the risk of bias. RevMan 5.4.1 (Review Manager 5.4.1; Cochrane Collaboration) software presented the results graphically.

### Statistical analysis

RevMan 5.4.1 and Stata 12.0 (StataCorp) software were used to analyze the data in this meta-analysis. Measurement data were used for statistical efficacy analysis using Cohen’s standardized mean difference (SMD) with 95% confidence intervals (CI). Cochran’s *Q* test and *I*^2^ statistics were used to examine overall heterogeneity between studies and within subgroups of studies. Benchmarks of *I*^2^ can be categorized as having low (25%), moderate (50%), and high (75%) heterogeneity (Higgins et al., 2003). Because of the variation of the study characteristics (e.g., mode of telephone intervention, participants’ characteristics), we assumed that the true effect size might vary from study to study. Thus, comparisons were based on a random-effects model (Borenstein et al., 2010). Media, strategy, group format, participant, and measurement time points subgroup analysis were used to examine the effect of different intervention types on loneliness outcomes in older adults. Three sensitivity analyses were performed to assess the stability of the pooled effects by omitting 1 of 3 individual studies to determine their influence on the pooled SMDs. Two studies (Shapira et al., 2007; Jarvis et al., 2019) were omitted because of the inadequate participant included in treatment, and the other (Tsai et al., 2020) was omitted because of its large CI. The remaining studies (the group with adequate participants included in treatment or the group with relatively small CIs) were then used to recalculate the pooled SMDs. A funnel plot was applied to detect publication bias. The Egger test evaluated the significance of the intercept. All *p* values were two-sided, with *p* < 0.05 considered significant.

## Results

### Literature screening

**FIGURE 1:**
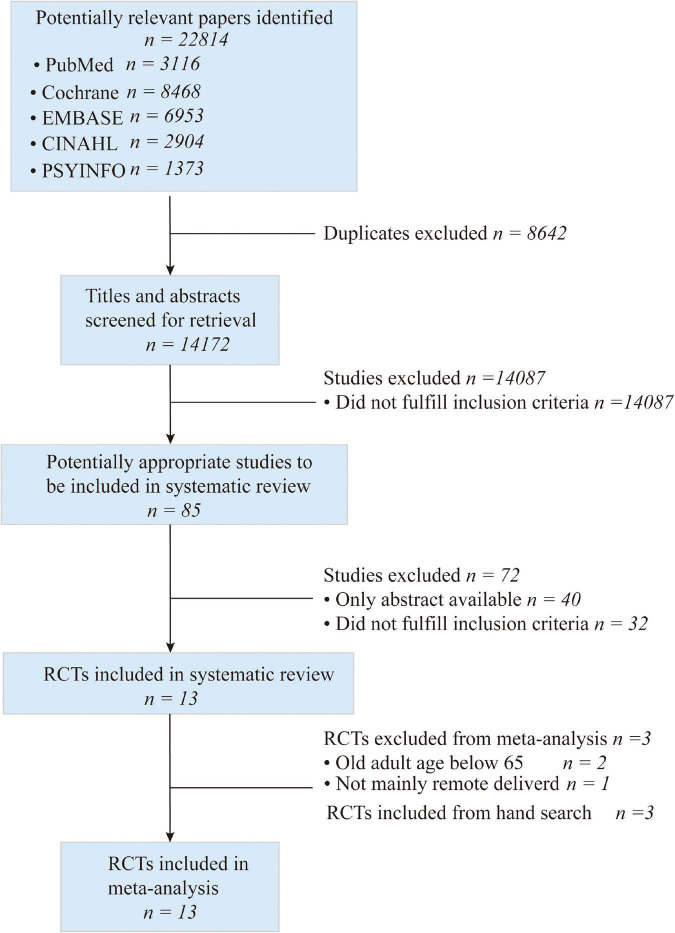
Flowchart of study selection.

As shown in Figure 1, 22,814 studies were identified with computerized search; after importing these articles into EndNote X9 software, 8,642 were duplicated articles, and 14,087 did not meet our inclusion of criteria following a review of the title and abstract. The full text of the remaining 85 articles was obtained. In addition, 75 studies were excluded for the following reason: conference abstract (*n* = 40), did not address loneliness and remotely delivered intervention (*n* = 32), age below 65 (*n* = 2; Heckman et al., 2006; Brodbeck et al., 2019), treatment not mainly through remotely delivered intervention (*n* = 1; Conwell et al., 2021). Finally, three studies were included in a hand search. Thirteen articles that met our inclusion criteria were included in the qualitative synthesis (Heller et al., 1991; Hartke and King, 2003; Shapira et al., 2007, 2021; Slegers et al., 2008; Mountain et al., 2014; Jing et al., 2018; Jarvis et al., 2019; Nelson et al., 2019; Choi et al., 2020; Lai et al., 2020; Tsai et al., 2020; Kahlon et al., 2021).

### Characteristics of the included studies

Thirteen RCTs that assessed subjects were included in the meta-analysis. Characteristics of the included studies are summarized in Table 1. These studies were from seven different countries and regions: Canada (*n* = 1; Lai et al., 2020), ISRAEL (*n* = 2; Shapira et al., 2007, 2021), Taiwan (*n* = 1; Tsai et al., 2020), United Kingdom (*n* = 1; Mountain et al., 2014), China (*n* = 1; Jing et al., 2018), South Africa (*n* = 1; Jarvis et al., 2019), United States (*n* = 6; Heller et al., 1991; Hartke and King, 2003; Nelson et al., 2019; Choi et al., 2020; Conwell et al., 2021; Kahlon et al., 2021). The included studies were published between 1991 and 2021, with sample sizes ranging from 32 to 294. Four studies were carried out in long term care (LTC; Shapira et al., 2007; Jarvis et al., 2019; Nelson et al., 2019; Tsai et al., 2020). Six studies used telephone call intervention (Heller et al., 1991; Hartke and King, 2003; Mountain et al., 2014; Nelson et al., 2019; Lai et al., 2020; Kahlon et al., 2021), three studies used video call intervention (Choi et al., 2020; Tsai et al., 2020; Shapira et al., 2021), four studies used the computer or internet-based intervention (Shapira et al., 2007; Slegers et al., 2008; Jing et al., 2018; Jarvis et al., 2019).

**TABLE 1:** Characteristics of the Included RCTs*.

Study	Location	Remotely delivered intervention group								Control group			
		Age (yr)	Men (%)	Media	Strategy	Group	Participant	Interval (h)/ duration (wk) /session	Sample size	Intervention	Sample size	Measurement timepoint (wk)	Outcome measure (loneliness)
Hartke and King, 2003	United States	70	26	Telephone	Support	Group	Caregiver	1/8/1	43	Usual care	45	24	UCLA
Lai et al., 2020	Canada	80	33	Telephone	Support	Group	Isolation	?/8/?	30	Brief contact	30	24	DJLS
Kahlon et al., 2021	United States	70	21	Telephone	Support	Indvdl	Isolation	0.2/4/5	120	Usual care	120	4	UCLA, DJLS
Heller et al., 1991	United States	74	0	Telephone	Contact	Group	Community	?/30/?	241	No treatment	53	10,20,30	UCLA
Shapira et al., 2021	Israel	72	19	Video call	Skills	Mixed	Isolation	2–3/4/?	64	No treatment	18	4	UCLA
Tsai et al., 2020	Taiwan	81	25	Video call	Contact	Group	LTC	0.1/24/1	32	No treatment	30	4,12,24	UCLA
Jarvis et al., 2019	South Africa	75	13	Internet	Cognition	Group	LTC	1.5/4/2	15	Usual care	17	2,4	DJGLS
Nelson et al., 2019	United States	76	47	Telephone	Cognition	Indvdl	LTC	0.8/7/1	31	Usual care	28	8,16	UCLA
Mountain et al., 2014	United Kingdom	82	34	Telephone	Contact	Mixed	Community	0.3/6/1	35	Usual care	35	24	DJGLS
Choi et al., 2020	United States	74	33	Video call	Cognition	Indvdl	Isolation	?/5/1	43	Brief contact	46	6,18	PROMIS-L
Jing et al., 2018	China	75	30	Internet	Cognition	Indvdl	Community	?/6/6	40	Soc activity	39	24	3-point Likert scale
Slegers et al., 2008	United States	70	?	Internet	Contact	Indvdl	Community	4/2/3	57	No treatment	38	16	DJGLS
Shapira et al., 2007	Israel	80	41	Internet	Contact	Indvdl	LTC	?/15/1	22	Soc activity	26	14	UCLA

UCLA, UCLA loneliness scale, University of California at Los Angeles; DJLS, De Jong Loneliness scale; DJGLS, The De Jong Gierveld Short Scales for Emotional and Social Loneliness; and PROMIS-L, Patient-Reported Outcomes Measurement Information System-Loneliness.

*RCT, randomized controlled trial; Soc activity, social activity; LTC, Long term care; and Indvdl, individua.

### Quality of the included studies

**FIGURE 2:**
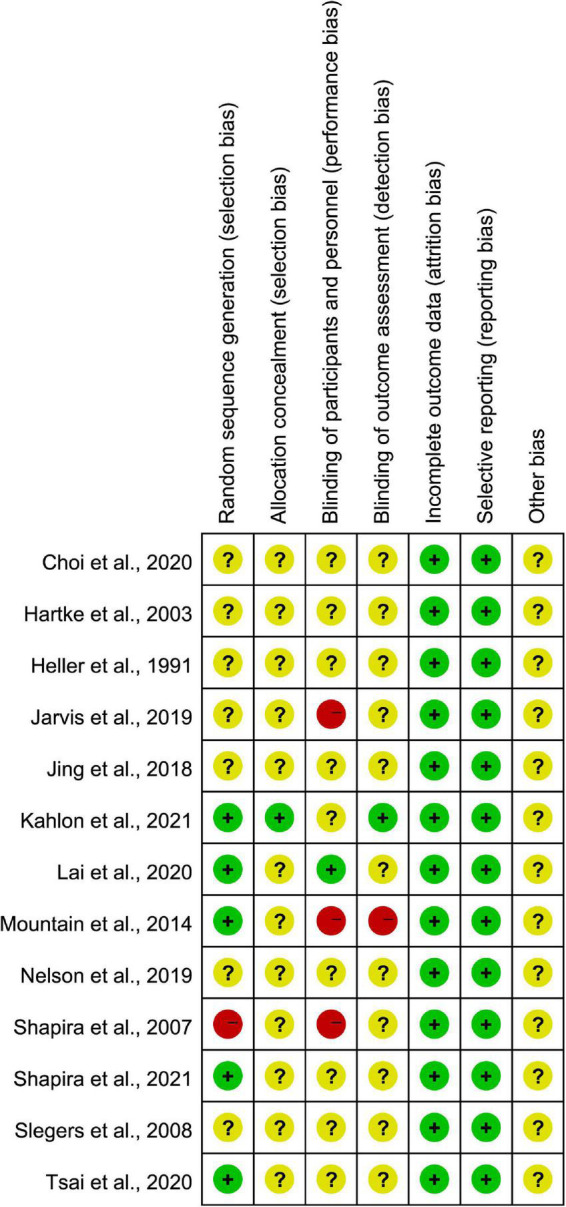
Validity of the included RCTs.

Adequate random allocation sequences were used in five studies (Mountain et al., 2014; Lai et al., 2020; Tsai et al., 2020; Kahlon et al., 2021; Shapira et al., 2021). One study inadequate randomization method (Shapira et al., 2007). The randomization methods of the other studies were unclear because the authors only mentioned that allocation was randomized in their studies. One study mentioned allocation concealment (Kahlon et al., 2021), which used envelopes. One study blinded the participants (Lai et al., 2020), and one blinded outcome assessment (Kahlon et al., 2021). Three studies did not use correct blinding methods (Shapira et al., 2007; Mountain et al., 2014; Jarvis et al., 2019). The result of the validity assessment is in Figure 2.

### Effect of remotely delivered intervention on loneliness in older adults

**FIGURE 3:**
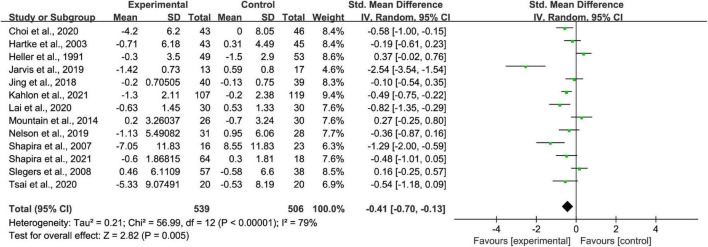
Comparisons of remotely delivered intervention and all controls on the basis of loneliness scores.

Thirteen studies were used to produce a random-effects model for loneliness. The remotely delivered intervention group had significantly better overall loneliness scores (*p* < 0.01; SMD = −0.41 [95% CI, −0.70 to −0.13]; *I*^2^ > 50%; Figure 3) than the control group (Figure 3). There was evidence of high heterogeneity among these studies since the *I*^2^ value is >50%.

### Subgroup analysis

#### Media subgroup analysis

**FIGURE 4:**
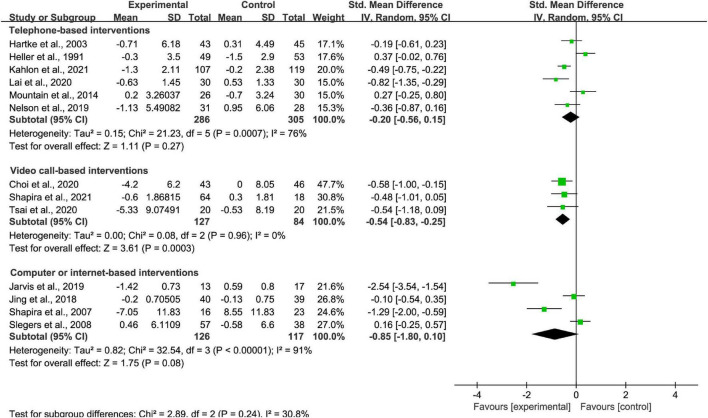
Subgroup analysis: comparison of telephone-based interventions, video call-based intervention or computer or internet-based interventions and all controls on the basis of loneliness scores.

Among the thirteen studies included, six were telephone-based interventions, three were video call-based intervention, and four were computer or internet-based interventions. It showed significantly superior video call- delivered intervention loneliness scores (*p* < 0.01; SMD = −0.54 [95% CI, −0.83 to −0.25]; *I*^2^ < 50%; Figure 4). No evidence was found to support the effective ness of telephone call and computer and internet- delivered intervention (*p* > 0.05; SMD = −0.20 [95% CI, −0.56 to 0.15]; *I*^2^ > 50% and *p* > 0.05; SMD = −0.85 [95% CI, −1.80 to 0.10]; *I*^2^ > 50%, respectively; Figure 4). There was high heterogeneity between studies of telephone-based interventions and computer or internet-based interventions, while there was no heterogeneity between studies of video call-based interventions.

#### Treatment strategy subgroup analysis

**FIGURE 5:**
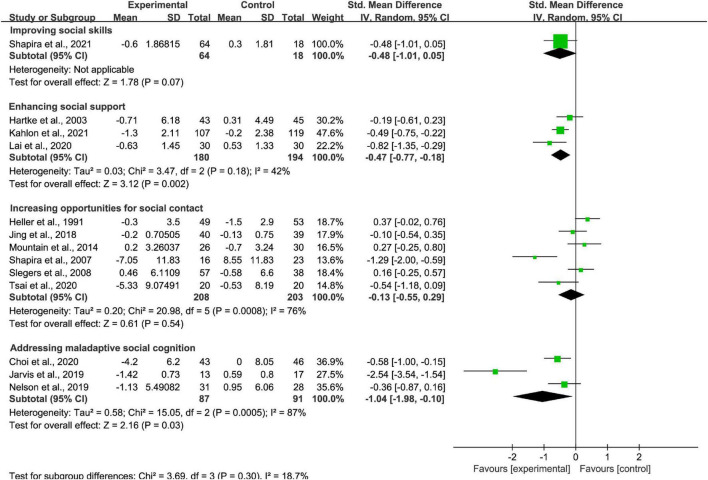
Subgroup analysis: comparison of improving social skill interventions, enhancing social support interventions, increasing opportunities for social contact interventions, or addressing maladaptive social cognition interventions and all controls on the basis of loneliness scores.

One study applied improving social skill interventions, three studies applied enhancing social support interventions, six studies applied increasing opportunities for social contact interventions, and three studies applied addressing maladaptive social cognition interventions. The effectiveness of Enhancing social support strategy and addressing maladaptive social cognition strategy (*p* < 0.01; SMD = −0.47 [95% CI, −0.77 to −0.18]; *I*^2^ < 50% and *p* < 0.05; SMD = −1.04 [95% CI, −1.98 to −0.10]; *I*^2^ > 50%, respectively; Figure 5) were noted. The effectiveness of improving social skill strategy and increasing opportunities for social contact strategy (*p* > 0.05; SMD = −0.48 [95% CI, −1.01 to 0.05] and *p* > 0.05; SMD = −0.13 [95% CI, −0.55 to 0.29]; *I*^2^ > 50%, respectively; Figure 5) were not found. There was high heterogeneity between studies of increasing opportunities for social contact interventions and addressing maladaptive social cognition interventions, nevertheless there was moderate heterogeneity between studies of enhancing social support interventions.

#### Participants subgroup analysis

**FIGURE 6:**
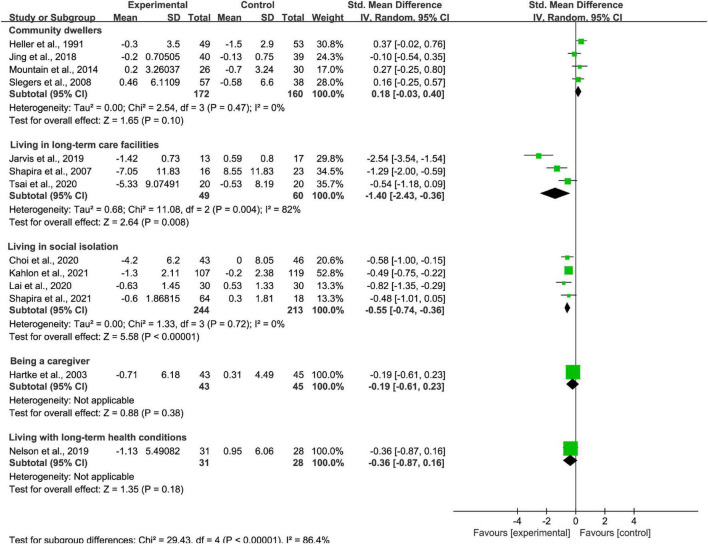
Subgroup analysis: comparison of old adults as community dwellers, living in long-term care facilities, living in social isolation, being a caregiver or living with long-term health conditions and all controls on the basis of loneliness scores.

Four studies included older adults as community dwellers, three studies included older adults living in long-term care facilities, four studies included older adults living in social isolation, one study included older adults being a caregiver, and one study included older adults living with long-term health conditions. It found significantly superior loneliness reduction for participants in social isolation and living in LTC settings (*p* < 0.01; SMD = −0.55 [95% CI, −0.74 to −0.36]; *I*^2^ < 50% and *p* < 0.01; SMD = −1.40 [95% CI, −2.43 to −0.36]; *I*^2^ > 50%, respectively; Figure 6). The effectiveness was not found for participants living as community dwellers (*p* > 0.05; SMD = 0.18 [95% CI, −0.03 to 0.40]; *I*^2^ < 50%; Figure 6), being a caregiver (*p* > 0.05; SMD = −0.19 [95% CI, −0.61 to 0.23]; Figure 6), and living with long-term health conditions (*p* > 0.05; SMD = −0.36 [95% CI, −0.87 to 0.16]; Figure 6). There was high heterogeneity between studies of interventions for older adults living in long-term care facilities, while there was no heterogeneity between studies of interventions for those as community dwellers and living in social isolation.

#### Group format subgroup analysis

**FIGURE 7:**
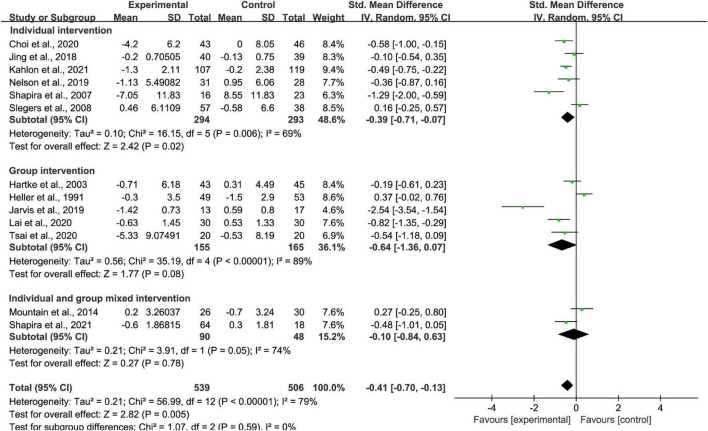
Subgroup analysis: comparison of individual interventions, group interventions, or mixed interventions and all controls on the basis of loneliness scores.

Among the included studies, six interventions were carried out in individual format, five interventions were carried out in group format, and two interventions were carried out in individual and group mixed format. When delivered individually, superior intervention loneliness scores (*p* < 0.05; SMD = −0.39 [95% CI, −0.71 to −0.07]; *I*^2^ > 50%; Figure 7) was discovered through the analysis. Intervention delivered in Group and mixed format showed no effective on loneliness reduction (*p* > 0.05; SMD = −0.64 [95% CI, −1.36 to 0.07]; *I*^2^ > 50% and *p* > 0.05; SMD = −0.10 [95% CI, −0.84 to 0.63]; *I*^2^ > 50%, respectively; Figure 7). There was high heterogeneity among these studies.

#### Measurement time points subgroup analysis

**FIGURE 8:**
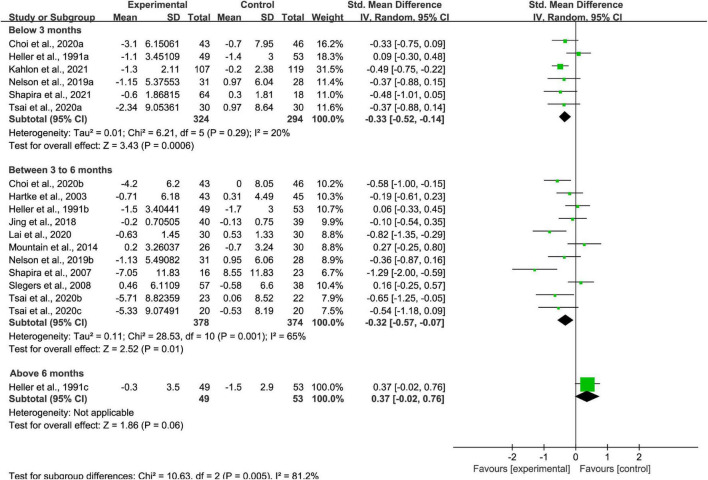
Subgroup analysis: comparison of interventions at measurement time points below 3 months, between 3 and 6 months, or above 6 months and all controls on the basis of loneliness scores.

For intervention effect measured at below 3 months stage, between 3 and 6 months stage, and above 6 months stage, superior loneliness scores were found at below 3 months stage (*p* < 0.01; SMD = −0.33 [95% CI, −0.52 to −0.14]; *I*^2^ < 50%; Figure 8) and three to 6 months stage (*p* < 0.01; SMD = −0.32 [95% CI, −0.57 to −0.07]; *I*^2^ > 50%; Figure 8). When the measurement time point is above 6 months, effectiveness of intervention on loneliness reduction did not exist (*p* > 0.05; SMD = 0.37 [95% CI, −0.02 to 0.76]; Figure 8). There was high heterogeneity between studies at measurement time points between 3 and 6 months, but there was low heterogeneity between studies at measurement time points below 3 months.

### Sensitive analysis

The sensitivity analyses revealed stable results (Table 2); excluding either of the three previously mentioned studies (Shapira et al., 2007; Jarvis et al., 2019; Tsai et al., 2020) did not alter the pooled SMDs.

**TABLE 2:** Sensitivity analysis (omitting a single RCT)*.

	Loneliness SMD (95% CI)
All studies	−0.32 (−0.44 to −0.19)
Selected study omitted	
Jarvis et al., 2019	−0.28 (−0.42 to −0.15)
Shapira et al., 2007	−0.29 (−0.41 to −0.15)
Tsai et al., 2020	−0.30 (−0.44 to −0.18)

*RCT, randomized controlled trial; and SMD, standardized mean differences.

### Publication bias analysis

**FIGURE 9:**
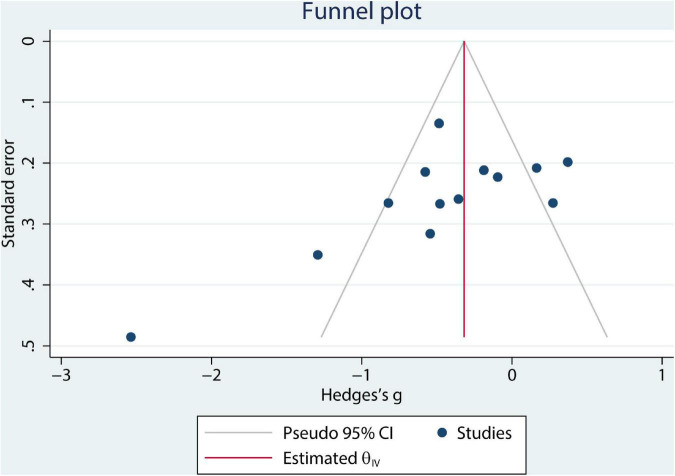
Funnel plot for overall studies.

Egger’s test and funnel plot was used to examine the publication bias of the included studies. The shape of the funnel plot shows asymmetry (Figure 9). Consistently, Egger’s test (*p* = 0.0004) suggested the result of the meta-analysis would be affected by publication bias.

## Discussion

This systematic review and meta-analysis demonstrate that remotely delivered intervention can result in loneliness reduction. The subgroup analysis suggested remotely delivered intervention had a superior effect on loneliness when delivered from an individual, by video call, using increasing social support or maladaptive social cognition treatment strategy, to older adults under LTC or social isolation circumstances, with measurement time points below 6 months, when compared with different control groups. These favorable effects of remotely delivered intervention involve complex interactions with the patient, including empathy, intention, care, and attention, that cannot be achieved by medications alone or by no intervention (Kahlon et al., 2021).

The previous meta-analyses have drawn various conclusions depending on the types of study design (Choi et al., 2012; Milner et al., 2015; Noone et al., 2020; Jin et al., 2021). A study showed that the effects of remotely delivered intervention were significantly superior to those of usual care (Choi et al., 2012). This result is in agreement with our findings.

The current study revealed new findings that differ from previous reports through subgroup analysis. First, when the effects of remote delivery methods on loneliness were quantitative compared simultaneously, video call-based intervention was superior to telephone-based intervention and computer or internet-based intervention when treating loneliness. In contrast, previous systematic reviews only qualitatively evaluated the effects of different methods of remote delivery on loneliness (Chen and Schulz, 2016; Gorenko et al., 2021). This study did not support the conclusion of a previous review that showed both video-call and telephone-based intervention would effectively reduce loneliness in older adults (Gorenko et al., 2021). The possible reason for the superior effect of video call-based intervention might be that video call-based intervention would give more social cues than the telephone and internet intervention, making participants feel more supported during the intervention (Noone et al., 2020).

Second, through quantitative comparison between four strategies on loneliness, this study found the effects of remotely delivered intervention addressing maladaptive social cognition and enhancing social support was better than those improving social skills and increasing opportunities for social contact when treating loneliness. In comparison, previous systematic reviews only examined the effectiveness of specific strategies on loneliness in remotely delivered intervention (Cattan et al., 2005; Chen and Schulz, 2016). The effectiveness of intervention addressing maladaptive social cognition (Masi et al., 2011) is supported in this study. However, the effects of intervention focus on social connectedness (Chen and Schulz, 2016) and opportunities for social contact (Cattan et al., 2005) are not supported. According to the cognition-biased model, the possible reason for the superior effect of an intervention addressing maladaptive social cognition and enhancing social support might be that social network effects on loneliness are mediated by social cognition (Larose et al., 2016). Thus, compared with increasing the ability or opportunity to enlarge the social network, changing cognition and giving feels of being socially supported might be more direct and effective for loneliness treatment in older adults.

Third, through a quantitative comparison of remotely delivered intervention on participants under different conditions, the research found the effects of remotely delivered intervention for participants living in social isolation and LTC settings were better than for community dwellers, caregivers, and those with chronic disease. In contrast, the previous studies only qualitatively examined the effectiveness of remotely delivered intervention for older adults in specific settings like LTC (Quan et al., 2020) or COVID-19 (Gorenko et al., 2021; Williams et al., 2021). The effectiveness of remotely delivered intervention for older adults in LTC settings (Quan et al., 2020) was confirmed in this study. Loneliness could be temporal or chronic. People with temporal loneliness are inclined to combat loneliness actively, while people with chronic loneliness are linked with helplessness and face loneliness passively (Perse and Rubin, 1990). The loneliness was temporal for the dwellers who transited to LTC settings or lived in social isolation caused by situation changes like COVID-19. They might find ways to combat transitional loneliness actively through remotely delivered intervention. However, as community dwellers, caregivers, and those with chronic diseases, the older adults might live alone for a long time, and their loneliness was chronic. They would be more passively facing the loneliness. Thus, it might be more effective for participants in temporal loneliness than chronic loneliness in front of the remotely delivered intervention.

Fourth, we found that remote intervention delivered individually was better than in a group through quantitative analysis. In comparison, previous research only qualitatively evaluated intervention effects with different group formats (Cohen-Mansfield and Perach, 2015; Poscia et al., 2018). The empathy of callers and their characteristics likely affected participants, which might increase the effectiveness of the treatment (Kahlon et al., 2021). Thus, intervention carried out in an individual format might be more effective.

Fifth, from the quantitative analysis result, the positive effect of remotely delivered intervention on loneliness seemed to be short-term. In contrast, the previous reviews only qualitatively examined and evaluated the effects of the intervention at different time stages (Chen and Schulz, 2016; Gorenko et al., 2021). This study supported the conclusion from a previous review published in 2016 that showed the positive effect of remotely delivered intervention on loneliness could last less than 6 months (Chen and Schulz, 2016). The positive effect could not last for a long time because the included RCT studies are directional treatment, which only focuses on maladaptive cognition change or social network enhancement. However, the loneliness of older adults could result from system reasons. Without combination with other possible solutions for loneliness treatment, like connector interventions, gateway approaches, and system approaches (Lee et al., 2021), the loneliness problem of older adults could only be partially resolved. Thus, the positive effects of remotely delivered intervention on loneliness cannot last long in older adults.

Finally, two high-quality RCTs have been included here for the first time. They showed significant effects of remotely delivered intervention compared with usual care or no treatment. By randomized clinical trial, Kahlon et al. (2021) found that remotely delivered intervention that was carried out by A layperson-delivered, empathy-oriented telephone call program has a significant effect on loneliness and depression. Shapira et al. (2021) used a pilot RCT design, and they found a significant improvement in the intervention group in terms of loneliness compared with the control group. Based on the above data, the findings of the two recent RCTs did support the effectiveness of remotely delivered intervention.

This meta-analysis has some limitations. First, the included RCTs compared various control interventions; therefore, definite conclusions regarding the various control interventions are not possible. Further evidence using large-scale, RCTs must be obtained to inform government and health providers about the efficacy of remotely delivered interventions. Second, to avoid the treatment provider empathy and characteristics effect, future studies should consider the patients’ attitudes regarding providers prior to treatment. Third, many studies consisted of older adults from western countries and have limited generalizability. Fourth, theoretical understandings of how successful interventions tackle loneliness are urgently needed. Finally, the lack of remotely delivered intervention protocols standardization also limits our findings.

In conclusion, we believe that remotely delivered intervention can provide superior loneliness relief than brief intervention, usual care, and no intervention. The effect on loneliness reduction appears to be affected by intervention technology, strategy, participants’ characteristic, group format, and effect measurement time point. This study highlights the value of remotely delivered intervention in reducing loneliness and warrants a broader usage investigation. These interventions may align with COVID-19 shielding/social distancing measures with minor modifications and help older adults tackle loneliness.

## Data availability statement

The original contributions presented in this study are included in the article/Supplementary material, further inquiries can be directed to the corresponding author.

## Author contributions

ZF designed the study, wrote the first draft of the manuscript, and supervised the manuscript production. ZF, MY, and CM performed the literature search, article selection, quality appraisal, statistical analysis, and participated in the revision of the subsequent draft. All authors read and approved the final manuscript.
